# Practitioners and Nursing Homes: A Suggested Reform

**Published:** 1913-11-08

**Authors:** 


### HOSPITAL WEAKNESS AND HOSPITAL STRENGTH.

**(Contributions to this page will be welcomed.)**

### Practitioners and Nursing Homes.

A SUGGESTED REFORM.

We welcome the new movement in the direction of the establishment of Pay Hospitals, which is manifesting itself in the Metropolis. This movement owes its origin to the abuses in nursing homes which have excited, with growing force, a spirit of rebellion amongst those classes of the community who are dependent upon the accommodation of nursing homes, when they have to undergo a serious operation. The complaints which have reached us from time to time, with regard to the absence of administrative control, deficiencies in the nursing organisation, the quality of the food, and the absence of continuous courtesy as a cardinal feature of these establishments, have made us wonder how long these unnecessary troubles would be borne in silence by the patients and their friends. Not only do such nursing homes acquire a reputation for defective administration, from the many abuses and discomforts which result, but the overcharges in many cases have become notorious. Such abuses are indefensible, for if the average nursing home was conducted on business principles by business people, most of the evils complained of would disappear as a matter of necessity. As matters stand at present, year after year in a number of homes, some of which frequently change hands, the administration shows little or no improvement, whilst the charges exhibit a growing tendency to increase.

### The Existing Abuses.

The remarkable fact is that abuses are not confined to the older nursing homes. We had an opportunity of seeing one of the latest of them, which was supposed to have been specially planned and built, for the purpose, by men of business, who believed there was money in it. A visitor who casually went through the home might be pleased with the decorative and other features it presented. But when visited by those who understood the working of such an establishment, and the essential features which should be found in the plan of any new building of the kind, they must have been struck with the evidence almost everywhere presented, that the first consideration had been to make every inch of available space produce as much as possible in the way of return, whilst such business considerations had not been tempered by knowledge, and so the work of the staff in the new building was greatly hampered at the cost of hygienic efficiency and comfort. The desire of the promoters was to have an up-to-date home, to make it popular, and so to secure a good return on their money. They, however, had not been guided by knowledge and experience, and so one of the newest establishments of the kind is full of instruction to the initiated, for it demonstrates what to avoid in such a building. A nursing home must have wide passages, to enable people readily to pass and patients to be removed to and from the operation theatre and elsewhere with the maximum of facility, to avoid various inconveniences and even danger which might otherwise result. In the home we have in mind the passages are so narrow as to make it actually difficult for two people to pass each other, and the moving of patients and the general work of the establishment are inevitably rendered difficult or impossible. The promoters, as we have said, having evidently little practical knowledge, had no apprehension of the grave faults the building exhibited, and as the site was restricted, there is no possibility of remedying them, and so the evil must go on until the home is killed by competition, or fails to yield an adequate profit.

This is not an occasion to dwell upon the large sums of money, representing hardly earned savings, which have been lost by trained nurses, who have yielded to an ambition to have a home of their own. No nursing home can fulfil the proper requirements of the medical profession which has not adequate funds behind it, is not administered by experts, and which does not put the welfare and comfort of the patients before all other considerations.

We have often written on the responsibility of silence which attaches to every officer, and especially to the medical officers who are responsible for the treatment of the patients, in hospitals in this country. That responsibility rests with even greater force upon the practitioners of all ranks, through whose introduction patients are placed in nursing homes. Any member of the medical profession who recommends a nursing home to a patient, or his friends, is morally bound to see that every hygienic precaution is taken in that particular establishment to safeguard the inmates, that every patient of his shall be protected from overcharges, and that in regard to the food and nursing everything shall be of the very best. As matters stand at present much mischief has been done to the reputation of the medical profession as a whole, owing to the non-observance of the rule, that no practitioner should take a fee from a patient who has been introduced to a nursing home through his instrumentality, without providing that the efficiency of such a home in every particular is of the highest and best.

### A Practical Reform.

Patients complain over and over again that they are over-charged, that they are underfed, that the nursing provision is inadequate, or objectionable, or inefficient, but the practitioner seldom or never raises his voice in the interest of his patients, but displays a disposition to ignore such matters, and to shelter himself under the plea that they are none of his business. It would be a good thing, and we are thoroughly convinced that it is a matter which should engage the attention of the Medical Council, that the various associations which have to do with the ethics and prestige of the medical profession should make provision for the hearing of the complaints of patients in regard to the matters we have just referred to, with a view to enforcing upon the practitioner concerned in each case a realisation of his responsibility should he have failed to fulfil it.

We believe that it would be an excellent rule that there should be a board or tablet, placed prominently in the entrance hall of every nursing home, recording the names of the practitioners who have regularly sent patients, so that every practitioner may know that in so acting he takes upon himself the responsibility of guaranteeing his patients that the administration, the nursing, the food, and the average of comfort shall be such as fully to promote their welfare, and that each inmate may with confidence regard his medical attendant as a natural protector against over-charging, faulty administration, and nursing. The time has come when the various evils which have made too many nursing homes in the Metropolis a by-word, should be taken severely in hand, so as to render it impossible for practitioners of reputation henceforth to allow their patients to enter any but those homes which are free from such abuses.

